# Increased cardiovascular mortality more than fifteen years after radiotherapy for breast cancer: a population-based study

**DOI:** 10.1186/1471-2407-7-9

**Published:** 2007-01-15

**Authors:** Rahul Roychoudhuri, David Robinson, Venkata Putcha, Jack Cuzick, Sarah Darby, Henrik Møller

**Affiliations:** 1 King's College London, Thames Cancer Registry, 1^st ^Floor Capital House, 42 Weston Street, London SE1 3QD, UK; 2 Centre for Epidemiology, Mathematics and Statistics, Wolfson Institute of Preventive Medicine, Charterhouse Square, London EC1M 6BQ, UK; 3 Clinical Trial Service Unit and Epidemiological Studies Unit, Radcliffe Infirmary, Oxford OX2 6HE, UK; 4 London School of Hygiene and Tropical Medicine, Keppel Street, London WC1E 7HT, UK

## Abstract

**Background:**

Breast radiotherapy as practised in the 1970s and 1980s resulted in significant myocardial exposure, and this was higher when the left breast was treated. It has been proposed that this difference might result in greater cardiovascular mortality following irradiation of the left breast when compared with the right.

**Methods:**

All cases of female breast cancer diagnosed between 1971 and 1988 and recorded on the Thames Cancer Registry database were followed up to the end of 2003 to identify cases who had died from ischaemic heart disease (IHD) or any cardiovascular disease (CVD). A proportional hazards regression analysis was performed, stratified by time since diagnosis, using as the baseline group those women with right-sided disease who did not receive radiotherapy, and adjusting for age at diagnosis.

**Results:**

A total of 20,871 women with breast cancer were included in the analysis, of which 51% had left-sided disease. Mortality at 15+ years after diagnosis was increased in recipients of left-breast radiotherapy compared to non-irradiated women with right-sided breast cancer, both for IHD (hazard ratio 1.59; 95% confidence interval 1.21–2.08; p = 0.001) and all CVD (hazard ratio 1.27; 95% confidence interval 1.07–1.51; p = 0.006). When irradiated women with left-sided breast cancer were compared with irradiated women with right-sided breast cancer, cardiovascular mortality at 15+ years after diagnosis was raised by around 25% (IHD: hazard ratio 1.23; 95% confidence interval 0.95–1.60; p = 0.114; CVD: hazard ratio 1.25; 95% confidence interval 1.05–1.49; p = 0.014).

**Conclusion:**

We have found an elevation in cardiovascular mortality more than 15 years after breast radiotherapy in women diagnosed with breast cancer between 1971 and 1988. The risk was greater following irradiation of the left breast compared with the right. This confirms that radiotherapy as practised in the 1970s and 1980s has resulted in significant long-term cardiac toxicity. In absolute terms, the increase in cardiovascular mortality induced by radiotherapy may be substantial, as these mortality events are relatively common.

## Background

Studies of survivors of atomic bomb irradiation reveal a radiation dose-related cardiovascular risk [1]. An overview of randomised trials on the effects of radiotherapy in breast cancer patients showed an excess mortality from circulatory diseases, and heart disease in particular [2]. However, the real impact of radiotherapy is likely to be underestimated in studies based on randomised control trials, because of the highly selected nature of trial participants, and it is important that such studies should be complemented by findings from population-based studies.

Breast radiotherapy as practised in the 1970s and 1980s resulted in significant myocardial exposure, and this was higher when the left breast was treated [3]. It has been proposed that this difference might result in greater cardiovascular mortality following irradiation of the left breast when compared with the right [3,4]. We have looked at cardiovascular mortality more than 15 years after diagnosis and treatment in women diagnosed with breast cancer in the 1970s and 1980s.

## Methods

All cases of female breast cancer diagnosed between 1971 and 1988 were extracted from the database held at the Thames Cancer Registry, a population-based register covering much of South East England. We excluded 3052 cancers of unknown laterality (4%) and 824 which were bilateral (1%). Deaths from breast cancer (ICD-9 174; ICD-10 C50), ischaemic heart disease (IHD) (ICD-9 410–414; ICD-10 I20-I25) or any cardiovascular disease (CVD) (ICD-9 390–459; ICD-10 I00-I99) were identified from death certificates.

Patients were considered at risk from the date of cancer diagnosis, but since death codes were not available prior to 1^st ^January 1997, only patients surviving beyond this date contributed to the analysis. Cases were followed up from 1^st ^January 1997 to 31^st ^December 2003 (or date of death if earlier), and were assumed to be alive at the latter time point if no notification of death had been received. Hence there were assumed to be no losses to follow-up. A proportional hazards regression analysis was performed, stratified by time since diagnosis, using as the baseline group those women with right-sided disease who did not receive radiotherapy, and adjusting for age at diagnosis. Further adjustments for tumour stage and socioeconomic status (based on the income score part of the Indices of Multiple Deprivation (IMD) [5]) had no significant effects on the estimated hazard ratios, and are not presented.

The study was based on routine cancer registration data, which are collected *inter alia *for the purpose of monitoring outcomes, and hence no ethical approval was required.

## Results

A total of 20,871 women with breast cancer were included in the analysis, of which 51% had left-sided disease. The mean age at diagnosis was 56.3 years (standard deviation 12.4 years). Tumour stage was not known in 23% of cases. Of those with known stage, two thirds were Stage 1 (localised) and 2.4% Stage 4 (metastases). 53% received radiotherapy. Although our data are insufficiently detailed to enable us to distinguish between different radiotherapy sites, we believe that the vast majority would have been to the breast or chest wall. Table 1 compares the characteristics of the four groups. There were significant differences across the groups in relation to mean age at diagnosis, deprivation quintile, stage and hormone therapy. However, when comparing left with right in patients with or without radiotherapy, there were no significant differences in any of these characteristics.

The median length of follow-up was 18.5 years, and was slightly shorter in the irradiated group (Table 1). During the period of observation, 1688 deaths from CVD (including 675 from IHD) were recorded, representing 8.1% and 3.2% of the study population respectively. Of these, 1023 and 426 respectively occurred more than fifteen years after diagnosis.

Table 2 shows age-adjusted hazard ratios (HRs) and the associated 95% confidence intervals (CIs) for deaths occurring 15 years or more after diagnosis. Mortality was increased in recipients of left-breast radiotherapy compared to non-irradiated women with right-sided breast cancer, both for IHD (HR 1.59; 95% CI 1.21–2.08; p = 0.001) and all CVD (HR 1.27; 95% CI 1.07–1.51; p = 0.006). There were no significant differences in breast cancer mortality between the groups. Those who received radiotherapy were younger and of higher socioeconomic status (SES), and had more advanced disease than the non-recipients (p < 0.001 in all cases). However, adjusting for stage and SES in addition to age made little difference to the estimated hazard ratios.

When irradiated women with left-sided breast cancer were compared with irradiated women with right-sided breast cancer, cardiovascular mortality at 15+ years after diagnosis was raised by around 25% (IHD: HR 1.23; 95% CI 0.95–1.60; p = 0.114; CVD: HR 1.25; 95% CI 1.05–1.49; p = 0.014).

## Discussion

We report a substantial elevation in cardiovascular mortality at greater than 15 years following radiotherapy to the left breast during the 1970s and 1980s. These results are consistent with the hypothesis that radiotherapy as practised during this period results in long-term cardiac toxicity, and confirm for the first time in a sample of UK patients the excess previously demonstrated in an American population [3].

Our findings are in general agreement with those of other long-term toxicity studies. A Swedish study covering the period 1970 to 1985 [6] found that breast cancer patients with left-sided tumours had significantly higher mortality due to myocardial infarction than patients with right-sided tumours. An overview based on randomised trials of radiotherapy initiated before 1975 [7] found an excess of cardiac deaths in patients surviving at least ten years. A more recent Dutch study [8] found that postmastectomy radiotherapy administered prior to 1979 and between 1979 and 1986 was associated with a 2-fold and 1.5-fold increase in cardiovascular mortality, respectively.

Our study has a number of shortcomings. We do not have detailed information on the radiation techniques involved, and it is possible that these may have changed during the period of the study. However, the lack of death details prior to 1997 make an analysis by period of treatment difficult, as period of treatment becomes aliased with period of follow-up. For the same reasons, we have not shown results for mortality at less than 15 years after diagnosis.

The comparison of left vs. right in the irradiated group used in our and other studies [3,4] is useful because therapy does not depend on laterality, and bypasses the potential problem of selection bias in a straight comparison of irradiated vs. non-irradiated women. As shown in Table 1, the differences in the observed characteristics between the irradiated and non-irradiated groups were eliminated when comparing left vs. right irradiated women. Whilst we were unable to obtain data on other cardiovascular risk factors such as smoking and exercise habits, it is unlikely that these would be related to laterality.

However, the exact quantification of the excess risk of cardiovascular mortality is not straightforward. A contrast between left and right sided breast cancer will tend to produce conservative estimates of risk because radiotherapy to the right breast, especially methods involving internal mammary lymph node (IMN) fields, also entails some cardiac dose.

In absolute terms, the increase in cardiovascular mortality that has been induced by radiotherapy may be substantial because these mortality events are relatively common. A woman who had breast cancer at the age of 50 and who survived to the age of 65 would, in the absence of radiotherapy, have a 22% risk of dying from cardiovascular disease in the next 20 years, up to the age of 85 (based on mortality and population data for England & Wales from National Statistics [9,10]). If the administration of radiotherapy increased this level of risk by 25% (as indicated by our left/right comparison), then the risk of cardiovascular death would increase by some 5 percentage points to a cumulative absolute risk of almost 30%. Moreover, mortality represents the extreme of the disease spectrum and is indicative of even higher levels of cardiovascular morbidity that do not always lead to death.

All of the patients in our study were treated for breast cancer in the 1970s and 1980s. During this period, techniques involving large fraction sizes, deep tangential fields or direct internal mammary fields were common. Since then, technical improvements in radiotherapy delivery have resulted in lower cardiac dosage, and recent studies [3,11] based on cancer registrations in the USA have shown a decline in the long-term risk of cardiac death after radiotherapy for breast cancer. More recent innovations such as conformal and intensity-modulated radiotherapy offer the potential of further reducing cardiac exposure [12].

## Conclusion

We have found a significant and important elevation in cardiovascular mortality at 15+ years following radiotherapy to the left breast. This result is consistent with the hypothesis that radiotherapy as administered in the 1970s and 1980s results in long-term cardiac toxicity.

## Competing interests

The author(s) declare that they have no competing interests.

## Authors' contributions

RR, DR and HM planned the study and drafted the manuscript. DR and VP analysed the data. JC and SD were involved in interpreting the results and critically revising the manuscript. All authors read and approved the final version.

## Pre-publication history

The pre-publication history for this paper can be accessed here:



## Figures and Tables

**Table 1:** Characteristics of study population by radiotherapy group and laterality

		**No radiotherapy**		**Radiotherapy**		**p- value**
		**Right**	**Left**	**Right**	**Left**	
**Age (years)**	Mean	58.1	58.4	54.5	54.8	< 0.001
	S.D.	13.2	13.2	11.6	11.4	
**IMD quintile**	1	1027 (21.9%)	1045 (20.8%)	1338 (24.6%)	1371 (24.0%)	< 0.001
	2	932 (19.9%)	1066 (21.2%)	1135 (20.9%)	1183 (20.7%)	
	3	890 (19.0%)	928 (18.4%)	1023 (18.8%)	1095 (19.2%)	
	4	979 (20.9%)	1072 (21.3%)	1007 (18.5%)	1042 (18.3%)	
	5	760 (16.2%)	843 (16.8%)	871 (16.0%)	936 (16.4%)	
	N/K	106 (2.3%)	78 (1.6%)	67 (1.2%)	77 (1.4%)	
**Stage**	1	2557 (54.5%)	2651 (52.7%)	2677 (49.2%)	2758 (48.4%)	< 0.001
	2	316 (6.7%)	331 (6.6%)	566 (10.4%)	553 (9.7%)	
	3	523 (11.1%)	554 (11.0%)	1016 (18.7%)	1071 (18.8%)	
	4	103 (2.2%)	105 (2.1%)	89 (1.6%)	92 (1.6%)	
	N/K	1195 (25.5%)	1391 (27.6%)	1093 (20.1%)	1230 (21.6%)	
**Chemotherapy**	No	4457 (95.0%)	4804 (95.5%)	5180 (95.2%)	5443 (95.4%)	0.602
	Yes	237 (5.0%)	228 (4.5%)	261 (4.8%)	261 (4.6%)	
**Hormone Therapy**	No	3445 (73.6%)	3785 (75.2%)	3904 (71.8%)	4127 (72.4%)	< 0.001
	Yes	1239 (26.4%)	1247 (24.8%)	1537 (28.2%)	1577 (27.6%)	
**Follow-up (years)**	Mean	20.31	20.46	19.26	19.19	< 0.001
	S.D.	5.45	5.50	5.02	4.97	
	Median	19.57	19.73	17.94	17.86	< 0.001

**Total cases**		4694	5032	5441	5704	

**Table 2:** Age-adjusted hazard ratios for deaths occurring 15 years or more after diagnosis of breast cancer

			**Time since diagnosis**			
**Death cause**	**Radio- therapy**	**Side**	**15+ years**			
			**Hazard Ratio**	**95% Confidence Interval**	**p-value**	**Deaths**
**Ischaemic Heart Disease**	No	R	1.00	-	-	93
		L	0.99	0.75 – 1.31	0.931	102
	Yes	R	1.28	0.96 – 1.70	0.092	101
		L	1.59	1.21 – 2.08	0.001	130
**All Cardiovascular Disease**	No	R	1.00	-	-	252
		L	0.97	0.82 – 1.15	0.747	271
	Yes	R	1.02	0.85 – 1.23	0.806	219
		L	1.27	1.07 – 1.51	0.006	281
**Breast Cancer**	No	R	1.00	-	-	208
		L	1.05	0.87 – 1.26	0.637	232
	Yes	R	1.05	0.87 – 1.27	0.600	229
		L	1.16	0.97 – 1.40	0.105	265
**All causes**	No	R	1.00	-	-	781
		L	1.05	0.95 – 1.15	0.359	896
	Yes	R	1.05	0.95 – 1.16	0.321	746
		L	1.16	1.05 – 1.28	0.003	856
